# Biomechanical properties of an all-soft tissue quadriceps tendon autograft compared with a quadrupled semitendinosus autograft: a cadaveric study

**DOI:** 10.1186/s43019-026-00337-1

**Published:** 2026-08-07

**Authors:** Thomas Sator, Lorenz Pichler, Gyula Kiss, Nicole Binder, Stephan Payr, Marcus Hofbauer, Thomas Koch, Sam Augustine Kandathil, Lena Hirtler, Thomas Manfred Tiefenboeck

**Affiliations:** 1 Department of Orthopedics and Trauma Surgery, Division of Trauma Surgery, Medical University of Vienna, Vienna, Austria; 2 Institute of Materials Science and Technology, TU Wien, Vienna, Austria; 3 Center for Anatomy and Cell Biology, Medical University of Vienna, Vienna, Austria

**Keywords:** ACL reconstruction, ACL biomechanics, Quadriceps tendon, Semitendinosus tendon, Adjustable loop device

## Abstract

**Background:**

Anterior cruciate ligament (ACL) injuries are among the most frequent ligament injuries, with autograft reconstruction considered to be the gold standard treatment. The all-soft-tissue quadriceps tendon (ASTQT) autograft has gained popularity because of its favorable graft size, reliable biomechanical strength, and reduced donor site morbidity. However, graft strength depends on the preparation, fixation, and construct stiffness. Emerging techniques, such as suture tape augmentation, may further influence biomechanical outcomes. The aim of this study was to investigate the biomechanical properties of tape-augmented ASTQT construct compared with quadrupled semitendinosus construct (QST) for anterior cruciate ligament reconstruction (ACLR) using an established cadaveric biomechanical test protocol.

**Methods:**

A total of nine human cadaveric ASTQTs and 11 QSTs were obtained from the knees of body donors. For the ASTQTs, both ends were secured using a suture tape-integrated adjustable loop suspensory fixation device. For the QSTs, the semitendinosus tendons were quadrupled and attached to two adjustable loops and stitched using the buried-knot technique described by Lubowitz. Cyclic biomechanical testing was performed following an established protocol, and load-to-failure, mode of failure, and stiffness were reported.

**Results:**

QSTs revealed a significantly higher load-to-failure (806.1 N versus 577.9 N, *P* < 0.001), initial (120.7 N/mm versus 93.3 N/mm, *P* < 0.001) and final (153.9 N/mm versus 117.1 N/mm, *P* = 0.001) dynamic stiffness and higher stiffness at failure (173.8 N/mm versus 120.1 N/mm, *P* < 0.001) compared with ASTQTs. All ASTQTs exhibited suture tape cut-through (cheese wiring) at the proximal fixation site as their mode of failure. Conversely, suture rupture was the predominant failure mode in the QST group.

**Conclusions:**

In an established cadaveric test setting, the QST construct exhibited higher load-to-failure and stiffness compared with the tape-augmented ASTQT construct. The consistent cheese-wiring failure at the proximal fixation site of the ASTQT indicates that the tape-tendon interface—not the substance itself—was the limiting structural element. These findings should be interpreted as construct-specific limitations of tape augmentation in degenerated tendon tissue, rather than as evidence of intrinsic inferiority of the quadriceps tendon graft.

## Introduction

Injuries to the anterior cruciate ligament (ACL) are the most common ligament injuries in the human body, with an incidence of approximately 70/100,000 cases [1]. The current gold standard treatment is arthroscopic ACL reconstruction (ACLR) with autografts, although the selection of the graft and fixation method is still a matter of debate [2, 3]. Hamstring tendons as quadrupled semitendinosus tendons or doubled semitendinosus-gracilis constructs and bone-patellar tendon-bone grafts are the most commonly used grafts for ACLR [4].

In recent years, the all-soft-tissue quadriceps tendon (ASTQT) graft has become increasingly popular for routine use in primary reconstruction of the ACL owing to its robust biomechanical properties, flexibility in graft size, and lower incidence of donor site complications [5–7]. Anatomically, the quadriceps tendon is thicker and wider than the hamstring tendons, providing substantial graft volume and mechanical stability, which has shown promising results in terms of patient-reported outcomes, revision rates, and surgical success in ACLR procedures [8–10]. Furthermore, harvesting the quadriceps tendon typically results in less postoperative pain and quicker rehabilitation compared with the BPTB graft [11]. Therefore, the ASTQT autograft is becoming a preferred choice for ACLR, particularly in adolescents and revision cases, offering a valuable alternative to traditional graft options [12].

When reconstructing the ACL, selection of a graft with properties closely resembling those of the native ACL is crucial. The tensile strength of the native ACL in young individuals has been reported to be approximately 2160 N, with maximum values reaching up to 2300 N. This strength declines markedly with age, decreasing to approximately 658 N in specimens older than 60 years [13, 14]. Comparative data indicate that quadriceps tendon autografts demonstrate ultimate load-to-failure values ranging from 249 to 2186 N, while hamstring tendon autografts range from 225 to 4590 N [6, 15, 16]. However, ultimate load-to-failure alone does not fully capture graft performance, as graft preparation techniques, fixation methods, and the overall construct stiffness critically influence outcomes—particularly in the context of accelerated rehabilitation protocols [12].

There are various techniques for the preparation of hamstring tendons in ACLR [17–19]. For the quadrupled semitendinosus tendon, the continuous loop technique and the buried knot technique by Lubowitz et. al. are well-known options [17]. Various preparation options are also available for the ASTQT. More recently, suture tape-reinforced preparation techniques have been described in which an integrated braided tape is used for additional support [20–23]. Independent tape augmentation functions as a secondary restraint—a “seat-belt mechanism”—that engages only after the graft has elongated beyond a threshold, thereby protecting the graft without sharing load during normal function [24, 25]. By contrast, integrated load-sharing configurations, in which the tape is stitched directly through the tendon substance and connected to the fixation device, result in simultaneous load transmission through both the tape and the tendon from the point of initial tensioning [26]. This latter configuration creates a composite construct where the tape-tendon interface becomes a potential site of mechanical failure, particularly in laminated tendon tissue. The biomechanical implications of such integrated load-sharing constructs have not been systematically evaluated in the literature [27].

Various graft fixation techniques have been described and investigated, including interference screws, suspensory cortical buttons, and hybrid constructs combining both methods. Particularly, the all-inside technique has gained popularity in recent years, allowing for precise graft placement, reduced bone removal, and less postoperative pain than traditional methods involving full tibial tunnels [10, 28–31]. In this technique, bony sockets are created with retrograde drills for graft passage and a double suspension fixation with adjustable loop devices (ALD) is used at both ends [24, 32].

Altogether, both the graft and fixation methods influence the stiffness of the entire construct, which plays a pivotal role in restoring native joint kinematics. Excessive stiffness can lead to elevated intra-articular chondral stress and may increase the risk of early-onset osteoarthritis [33, 34].

Despite numerous studies evaluating graft types and fixation strategies, the definitive clinical superiority of one approach over another remains unproven, underscoring the importance of biomechanical research in advancing ACL reconstruction techniques [12, 15].

The purpose of this study was to examine the biomechanical behavior of a tape-augmented all-soft tissue quadriceps tendon construct compared with a quadrupled semitendinosus construct for ACLR using a comprehensive human cadaveric model. We also wanted to document the failure modes of the constructs to identify the weakest link of both constructs.

We hypothesized that QST constructs would demonstrate superior load-to-failure and stiffness properties compared with tape-augmented ASTQT constructs in an experimental, biomechanical setting using an established test protocol.

## Methods

This study was carried out in compliance with the principles outlined in the Declaration of Helsinki and approved by the Ethics Committee of the  Medical University of Vienna (Ethics Commission Code: 2086/2019).

### Specimens

**Fig. 1:**
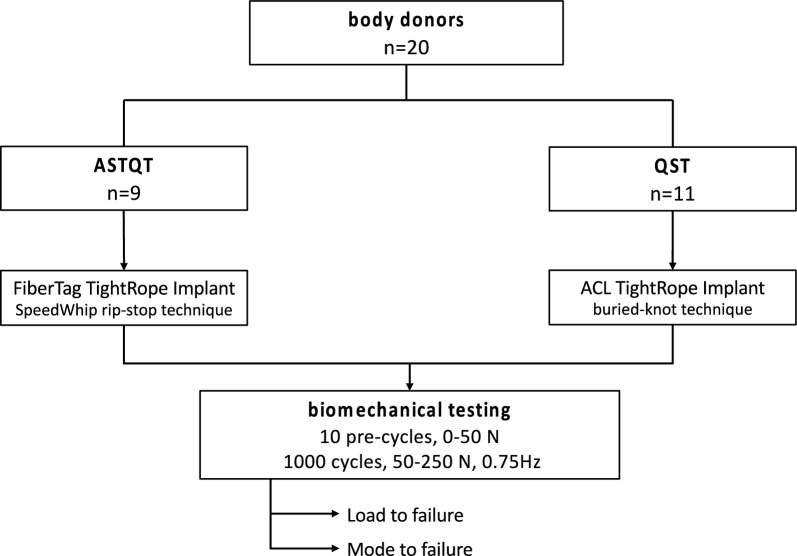
Flowchart of patient inclusion and overview of general testing procedure

Biomechanical testing was performed using an all-inside ACL reconstruction technique with human ASTQT and QST grafts. Specimens were checked for severe knee injuries or indications for surgery and were excluded and replaced before harvesting. A total of nine quadriceps tendons and 11 semitendinosus tendons were harvested from fresh frozen cadaveric knees at the  Center for Anatomy and Cell Biology, Medical University of Vienna, with a mean donor age of 80.2 years. The unequal group sizes resulted from specimen availability and application of exclusion criteria. Two additional quadriceps tendon specimens were harvested but excluded prior to testing owing to macroscopic signs of severe tendon degeneration that did not meet inclusion standards, whereas all harvested semitendinosus specimens met the quality criteria (Fig. 1). The ASTQT group was obtained from body donors with a mean age of 79.9 years and comprised four female and five male specimens. Conversely, the QST group was harvested from donors with a mean age of 80.5 years, including seven female and four male body donors.

### Graft preparation

**Fig. 2:**
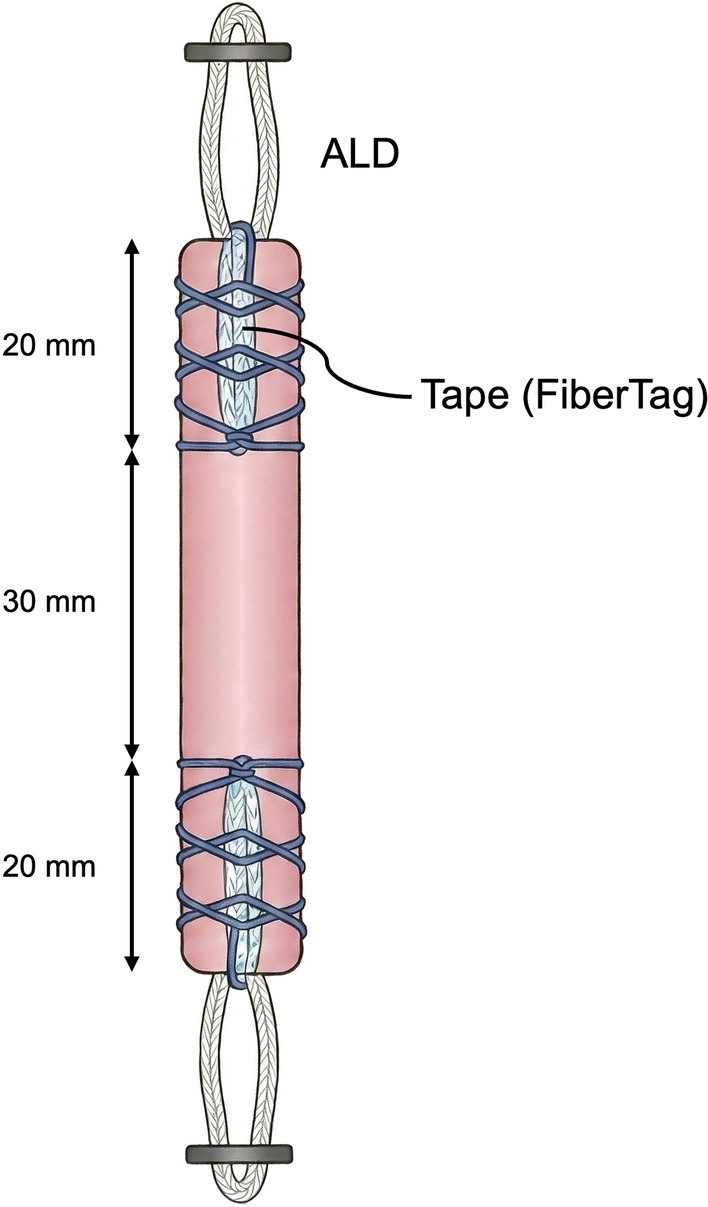
Schematic representation of the ASTQT graft construct. The graft is prepared to a total length of 70 mm. The FiberTag suture tape is integrated into both the proximal and distal end of the tendon. Circumferential stitches are passed through both the graft tissue and the tape. An adjustable loop device (ALD) is secured to both ends of the tendon

Specimens were thawed at room temperature for a minimum of 2 h before preparation and testing and were kept moist with physiological saline solution. The ASTQT graft was harvested in a full-thickness manner with a 9 mm double-blade knife and QT harvester (both Arthrex, Naples, FL, USA) to a length of 70 mm from the central aspect of the quadriceps tendon. For the ASTQT group, graft preparation was performed using the QuadLink system with FiberTag TightRope implants (Arthrex, Naples, FL, USA). This system utilizes a suture tape (FiberTag) that is integrated into the adjustable loop device. The FiberTag was incorporated into both ends of the graft using the SpeedWhip rip-stop technique. Six circumferential stitches were made through both the tendon tissue and the integrated tape on each side. Crucially, this stitching creates a construct where the FiberTag functions as a load-sharing augmentation. (Fig. 2) The QST graft was harvested by using a tendon stripper. Preparation of the tendon was carried out with the ACL TightRope® RT Implant (with preloaded ALD) using the buried-knot technique described by Lubowitz et al. in 2012. All tendons were assessed by a senior orthopedic surgeon (T.M.T) after harvesting. There were no signs of damage affecting the tissue quality. Before testing, all grafts were pretensioned for 5 min on a graft preparation board at 50 N.

### Test setup and protocol

Testing was conducted at a mechanical engineering laboratory under the guidance of a senior scientist with over two decades of experience in material testing. A calibrated universal material testing machine (Zwick Z050, ZwickRoell, Ulm, Germany) equipped with a force sensor was used. The testing protocol and setup were adapted from a previously described protocol [35]. The grafts were pretensioned to 50 N using the graft preparation board by Arthrex Naples, FL, USA, prior to being loaded onto the testing machine. Because the FiberTag suture tape was integrated into the tendon the graft and tape were tensioned simultaneously as a single composite construct.

**Fig. 3:**
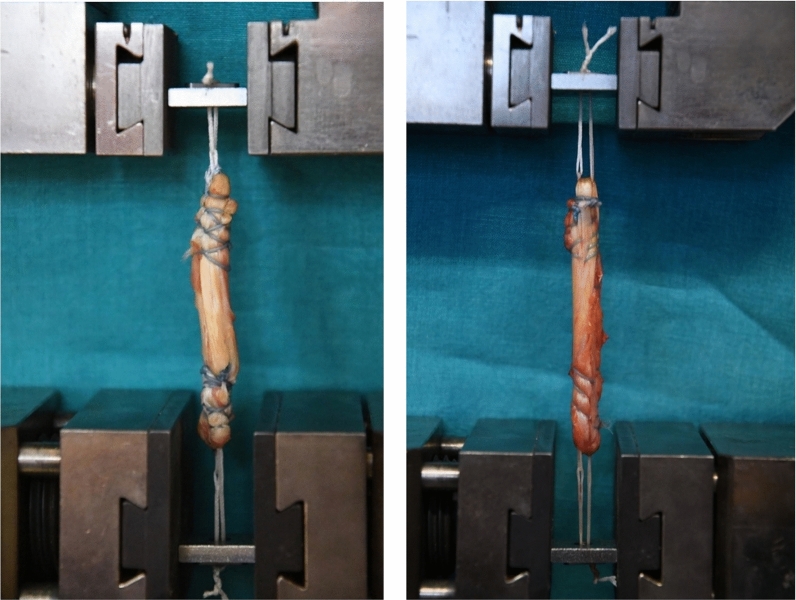
Experimental setup for biomechanical testing of the all-soft tissue quadriceps tendon (left) and quadrupled semitendinosus tendon graft (right), both with double-suspensory fixation on an electromechanical testing machine

Both grafts were fixed with the buttons of the ALD on a metal plate simulating the cortical bone. For ASTQT, the proximal portion of the graft was attached to the upper plate of the testing machine. The machine data output was recorded using ZwickRoell firmware (Fig. 3).

**Fig. 4:**
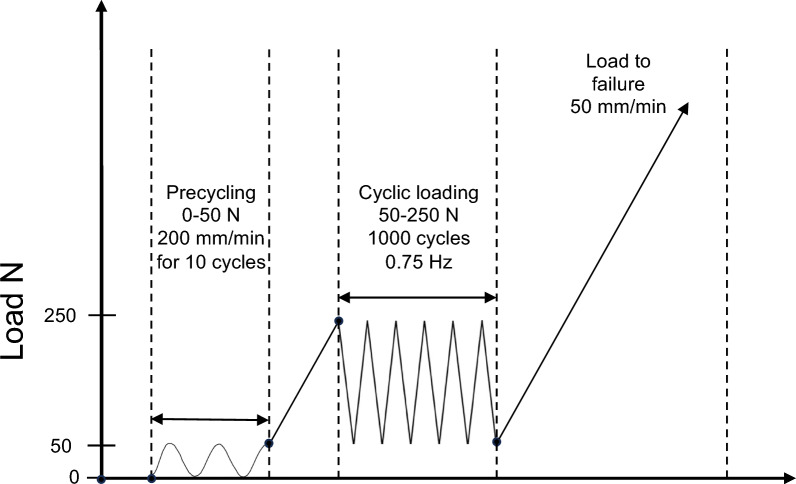
Testing protocol with points of data analysis. Metrics for comparison included initial and final dynamic stiffness, ultimate load, and stiffness during pull-to-failure

Biomechanical testing began with 10 pre-cycles, during which the graft construct was loaded with a force of 50 N at a speed of 200 mm/min to simulate the tension of the sutures tied over a cortical bone bridge. Following the preloading, cyclic force-controlled testing was conducted, consisting of 1000 cycles between 50 and 250 N at a frequency of 0.75 Hz (Fig. 4). The force applied during testing corresponds to the weight-bearing ACL forces occurring in vivo [36, 37], and is comparable to other studies in terms of peak load [35, 38–40]. Following cyclic testing, the load-to-failure was determined by increasing the tensile force at 50 mm/min until the graft failed. The force applied before resistance was lost was measured, and the failure mechanism was noted.

### Outcome data

Initial and final dynamic stiffness were calculated during the cyclic-testing phase. Dynamic stiffness (N/mm) was defined as the linear slope generated between the valley (50 N) and peak (250 N) loads in a single cycle. At the end of each 1000th cycle, the ultimate failure stiffness, ultimate load-to-failure, and mode of failure were described. The ultimate failure stiffness was determined during the load-to-failure testing, which was calculated as the linear slope within the load range of 200 N and 450 N.

### Statistical analysis

Categorical data were tested using either the Chi-square test or Fisher’s exact test. Differences in continuous variables were compared using Student’s *t*-test if they corresponded to a normal distribution. The Mann–Whitney *U*-test was performed where the results were not normally distributed. Normal distribution was checked using the Shapiro–Wilk test. In all analyses, a *P* < 0.05 was considered statistically significant. The sample size was based on an a priori power analysis (*α* = 0.05, *β* = 0.20) using data from the literature, which resulted in a minimum sample size of four. The obtained data were analyzed using Microsoft Excel^®^, SPSS^®^ Version 23 (IBM Corp., Armonk, N.Y., USA) and GraphPad Prism^®^ Software (GraphPad Software Inc., San Diego, CA, USA).

## Results

A total of 20 tendons were harvested (9 quadriceps tendons and 11 semitendinosus tendons) and later tested. After preparation, the mean ASTQT graft length was 6.89 cm (SD ± 0.70), with a mean graft diameter of 10.3 mm. The mean donor age in the QST group was 80.5 years. The mean length of the QST graft was 6.20 cm (SD ± 0.60). Mean graft diameter was 8.77 mm. Details regarding body donors and graft specifications are presented in Table 1.

**Table 1:** Body donor- and graft-specific data

	ASTQT	QST
Specimens, *n*	9	11
Sex		
Female, *n*	4	7
Male, *n*	5	4
Mean age, years (range, SD)	79.9 (57–90, 10.4)	80.5 (54–92, 13.5)
Side		
Right, *n*	4	7
Left, *n*	5	4
Graft length, cm (range, SD)	6.89 (6.0–8.0, 0.70)	6.20 (5.30–7.10, 0.60)
Graft diameter, mm (range, SD)	10.3 (9.50–10.5, 0.40)	8.77 (7.50–11.0, 0.94)

The ASTQT construct achieved lower initial (93.3 versus 120.7 N/mm, *P* < 0.001) and final (117.1 versus 153.9 N/mm, *P* = 0.001) dynamic stiffness in contrast to the QST construct. All the biomechanical outcome parameters are presented in Table 2.

**Table 2:** Biomechanical properties (stiffness and ultimate load-to-failure) of ASTQT and QST constructs

Outcome	ASTQT	QST	*P*-value
Initial dynamic stiffness, N/mm (range, SD)	93.3 (80.4–110.2, 9.90)	120.7 (96.4–151.6, 17.1)	< 0.001*
Final dynamic stiffness, N/mm (range, SD)	117.1 (102.7–132.4, 10.6)	153.9 (124.1–198.3, 23.9)	0.001*
Ultimate failure stiffness, N/mm (range, SD)	120.1 (108.0–138.1, 10.0)	173.8 (130.5–218.7, 30.2)	< 0.001*
Ultimate load-to-failure, N (range, SD)	577.9 (409.0–768.0, 100.6)	806.1 (541.0–975.0, 127.0)	< 0.001*

^*^Mann–Whitney-*U* test

**Fig. 5:**
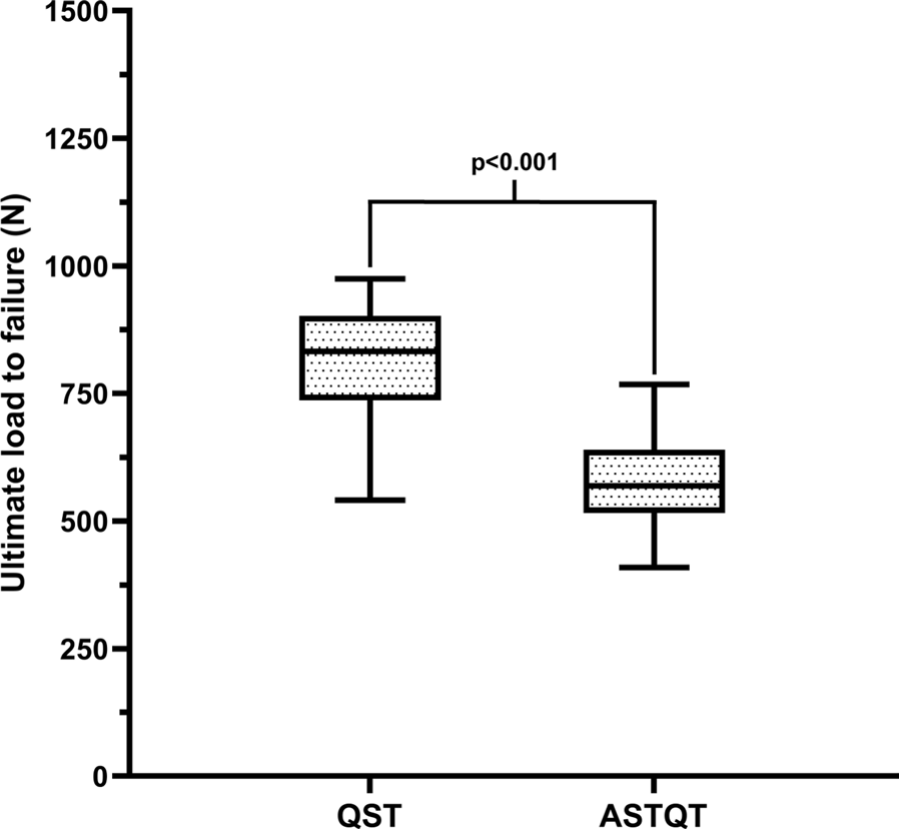
Ultimate load-to-failure testing for both test groups. *QST* Quadrupled Semitendinosus Tendon, *ASTQT* All-soft Tissue Quadriceps Tendon

**Fig. 6:**
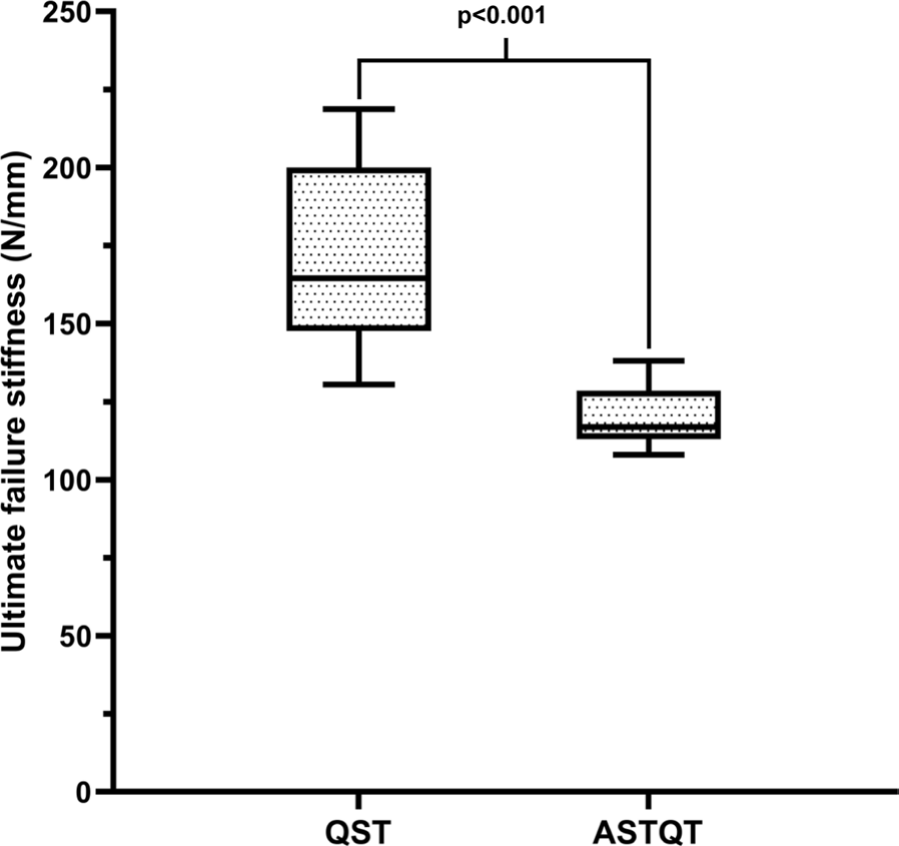
Ultimate failure stiffness for both test groups. *QST* Quadrupled Semitendinosus Tendon, *ASTQT* All-soft Tissue Quadriceps Tendon

**Fig. 7:**
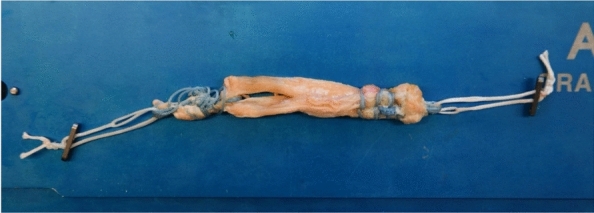
All-soft tissue quadriceps tendon construct after testing with suture tape cutting through the tendon at the proximal fixation site

ASTQT showed an ultimate load-to-failure of 577.9 N (SD ± 100.6), which was inferior to the QST group with 806.1 N (SD ± 127.0) (Fig. 5). Ultimate failure stiffness of the ASTQT remained below the level recorded in the QST group (120.1 versus 173.8 N/mm, *P* < 0.001) (Fig. 6). Regarding the mode of failure, cheese wiring was consistently observed in the ASTQT group, with the suture tape cutting through the tendon at the proximal fixation site in all nine specimens (Fig. 7). Failure occurred exclusively at the tape-tendon interface rather than within the tendon substance itself. For QST, suture failure was identified in 10 cases (90.9%) and button breakage in 1 case (9.1%).

## Discussion

The most important finding of this study was that the QST construct demonstrated superior biomechanical properties compared with the tape-augmented ASTQT construct in this specific cadaveric model. Stiffness and ultimate load-to-failure were significantly higher in the QST group.

The existing biomechanical literature on quadriceps versus hamstring grafts is mixed. Some studies report comparable properties when isolated tendons are tested, while others describe differences that vary with graft preparation and fixation [41–44]. Construct stiffness and ultimate load-to-failure are crucial factors, as they determine the capacity of the graft to withstand the demands of early rehabilitation and return to sports [41].

In general, both graft types showed lower ultimate load-to-failure and stiffness levels than those reported in recent studies [6, 16]. However, previous studies have only evaluated the tendons themselves without considering the effects of additional graft preparation techniques. In our study, we compared two surgical constructs—a tape-augmented ASTQT and a nonaugmented QST—rather than two graft tissues in isolation. The ASTQT construct included an integrated suture tape that shared the load with the tendon from the moment of initial tensioning, whereas the QST construct relied solely on suture fixation without tape augmentation. Therefore, the observed biomechanical differences reflect the combined effects of tissue properties, preparation technique, and fixation configuration. Readers should interpret the findings as construct-specific: the data demonstrate that the tape-augmented ASTQT configuration tested here exhibited inferior biomechanical properties compared with the QST configuration but do not permit conclusions regarding the intrinsic biomechanical potential of the quadriceps tendon graft itself.

This construct-specific interpretation is supported by the anatomical architecture of the two tendons. The quadriceps tendon is a laminated structure composed of parallel collagen sheets stacked along the transverse axis and is prone to interlaminar cleavage, particularly when degenerated or fatty-infiltrated with advancing age [2, 16]. By contrast, the semitendinosus tendon is a compact tubular structure with circumferentially organized collagen bundles that resist transverse cut-through, even under high suture tension.

When compared with studies using similar construct configurations, our findings align with existing literature. In a study that had the same setup with a QST and two ALDs, Madail et al. reported stiffness and load-to-failure values similar to our findings [45]. For the tape-augmented ASTQT, Lamplot et al. reported an ultimate load-to-failure of 637 N, which is higher than the 577.9 N we observed in our study. Also, the reported ultimate failure stiffness of 171.9 N/mm is higher than the 120.1 N/mm we found [46]. However, the donors examined in the previous study were significantly younger, with a mean age of 49.3 years (range: 28–68 years), compared with the 80.5 years (range: 54–92 years) for the ASTQT group in our investigation.

Studies testing isolated tendon constructs without tape augmentation or suspensory fixation have reported comparable ultimate load-to-failure between quadriceps and multi-strand hamstring grafts, with the hamstring construct generally demonstrating greater stiffness [43, 47]. The substantially lower values of the ASTQT construct in our cohort compared with those in the isolated tendon studies are therefore most consistent with an interaction between the tape-augmented construct design and age-related tendon degeneration rather than an intrinsic inferiority of the quadriceps tendon graft.

The failure pattern observed in this study supports this interpretation. All cheese-wiring failures occurred exclusively at the proximal fixation site of the quadriceps tendon. No failures were observed at the distal fixation, within the tendon mid-substance, or at the ALD-button interface. This failure mode represents a limitation of the tape-tendon interface under load-sharing conditions and not a failure of the tendon substance itself.

Lamplot et al. also described suture pullout at the proximal tendon as the predominant failure mode [46]. The geometry of our load-sharing augmentation construct explains this consistency: because the high-tensile tape and the degenerated, laminated tendon share load simultaneously, stress concentrates at the suture-tendon interface, causing the tape to cleave the tendon layers before the ultimate strength of the tape itself is reached [46, 48]. This interface failure is further supported by graft dimensions. Although the ASTQT grafts possessed a larger mean diameter (10.3 mm) compared with the QST grafts (8.77 mm), they demonstrated significantly lower ultimate load-to-failure. If the tendon substance had been the limiting factor, one would expect the larger-diameter construct to carry greater load, not less. This discrepancy further indicates that the limiting factor for the ASTQT construct was not the mid-substance strength or volume of the tendon itself but rather localized tissue failure at the proximal fixation.

The reported forces acting on the ACL during various movements range from 20 to 600 N [49]. For instance, the ACL experiences forces of 150 N during walking, 303 N during a single-leg stance, 445 N when descending stairs, and 450 N during jogging [37, 50]. Noyes et al. determined that the initial fixation strength of an ACL graft should exceed 450 N to ensure sufficient knee stability during daily activities [51]. Notably, the graft constructs in our study significantly exceeded this threshold, which is crucial for successful early rehabilitation after ACL reconstruction.

Beyond ultimate load, construct stiffness is a second key biomechanical determinant. In young individuals, the native ACL exhibits a mean stiffness of 220 ± 72 N/mm, with a progressive decline observed with advancing age [13, 14]. In the present study, the QST construct demonstrated stiffness values comparable to those of the native ACL, despite the advanced age of the donor specimens. By contrast, the ASTQT construct demonstrated a lower stiffness of 120.1 ± 10 N/mm, consistent with the previously reported mean stiffness of 124 ± 16 N/mm in older specimens, as described by Woo et al. [13, 14].

Since tendon stiffness decreases with age, it is plausible that in younger patients both grafts would demonstrate increased stiffness, with the QST potentially exceeding the physiological stiffness of the native ACL and the ASTQT graft construct approaching that of a young native ACL. Supporting this notion, Lamplot et al. reported a mean graft stiffness of 171.9 N/mm in specimens approximately 50 years of age under similar experimental conditions [46]. It is important to note that remodeling, maturation, and incorporation of the graft may further influence its biomechanical properties, which were not considered within the scope of this investigation [52]. Accurately replicating the mechanical behavior of the native ACL remains challenging but is of critical importance. While not directly measured in this study, theoretical models suggest that creating graft stiffness that significantly exceeds physiological levels could result in knee over-constraint and increased stress on the articular cartilage [12, 33, 34]. Future research in younger specimens is required to determine if the high stiffness of QST constructs translates to clinical over-constraint or early-onset osteoarthritis.

The selection of the optimal graft for anterior cruciate ligament surgery continues to be a topic of debate, as there is no universally recognized superior option available. Every graft presents both advantages and disadvantages, and graft choice is influenced by a combination of patient-specific factors—such as age, activity level, revision status and tissue quality—as well as surgeon preference and experience. Within the present study, effort has been made to find more answers regarding optimal graft choice using the all-inside surgery technique. However, the clinical applicability of these findings warrants further consideration. ACL reconstruction is predominantly performed in patients aged 15–40 years, whereas our specimens were harvested from donors with a mean age of approximately 80 years [1, 53, 54]. The age-related lamination and fatty degeneration of the quadriceps tendon that underlies the observed cheese-wiring failure mode may not occur to the same extent in younger, nondegenerated tissue. Nevertheless, the construct-specific limitations described here carry direct clinical relevance for patients in whom tendon quality may be compromised—such as older patients, revision scenarios, or individuals with preexisting tendinopathy—and may actively guide surgeons toward alternative fixation strategies or graft selection in these higher-risk settings. Further studies are needed to understand how different techniques and implants for graft fixation may impact overall graft performance and long-term clinical outcomes across these distinct patient populations.

This study had several limitations. As a cadaveric investigation, it represents a static, time-zero scenario that cannot account for the dynamic healing process and its potential influence on biomechanical properties. The advanced mean age of the body donors is a significant confounder, disproportionately affecting the laminated quadriceps tendon compared with the rope-like semitendinosus tendon, and likely predisposed the ASTQT group to the cheese-wiring failure mode observed. Furthermore, this study compares two distinct surgical constructs rather than two graft tissues in isolation: the asymmetric use of tape augmentation in the ASTQT group represents an inherent confounding factor that limits the ability to draw graft-specific conclusions. It is also important to recognize that the testing protocol used in this study represents a simplified representation of the complex forces present in the knee joint. The use of frozen cadaveric specimens may have also affected the biomechanical characteristics of the tissues, as the freezing and thawing cycles can potentially modify the structural and functional properties of the tendons. The limitations of this study must be considered when evaluating the relevance of these findings to clinical practice.

## Conclusions

In this geriatric cadaveric model, the QST construct demonstrated superior biomechanical properties compared with the tape-augmented ASTQT construct, with significantly greater stiffness and higher ultimate load-to-failure. The inferior performance of the ASTQT construct appears primarily related to failure at the suture tape-tendon interface rather than to intrinsic tendon properties: all failures occurred at the proximal fixation site through cheese-wiring of the laminated, degenerated tendon layers, while the mid-substance tendon strength was never the limiting factor. These findings should therefore be interpreted as construct-specific limitations of tape augmentation in elderly tendon tissue, rather than as evidence of a fundamental inferiority of the quadriceps tendon graft itself. Clinically, the results highlight that the biomechanical integrity of tape-augmented constructs depends on the suture-holding capacity of the native tendon tissue, and raise caution regarding the use of integrated load-sharing tape augmentation in clinical settings with potentially compromised tendon quality—such as older patients, revision scenarios, or preexisting tendinopathy. Direct extrapolation of these findings to the typical young ACL reconstruction population is limited, and future research in younger specimens with intact tendon architecture is required to establish the biomechanical behavior of these constructs in the intended patient group.

## Data Availability

The data that support the findings of this study are available on request from the corresponding author. The data are not publicly available owing to privacy or ethical restrictions.

## Abbreviations

ACL: Anterior cruciate ligament
ACLR: Anterior cruciate ligament reconstruction
ASTQT: All-soft tissue quadriceps tendon autografts
QST: Quadrupled semitendinosus autografts
ALD: Adjustable loop devices
